# Do Menopause and Aging Affect the Onset and Progression of Rheumatoid Arthritis and Systemic Lupus Erythematosus?

**DOI:** 10.7759/cureus.10944

**Published:** 2020-10-14

**Authors:** Lisa Shah, Abeer O Elshaikh, Robert Lee, Christopher Joy Mathew, Merin Tresa Jose, Ivan Cancarevic

**Affiliations:** 1 Family and Community Medicine, California Institute of Behavioral Neurosciences & Psychology, Fairfield, USA; 2 Internal Medicine, California Institute of Behavioral Neurosciences & Psychology, Fairfield, USA; 3 Surgery, California Institute of Behavioral Neurosciences & Psychology, Fairfield, USA; 4 Medicine, California Institute of Behavioral Neurosciences & Psychology, Fairfield, USA; 5 Family Medicine, California Institute of Behavioral Neurosciences & Psychology, Fairfield, USA

**Keywords:** rheumatoid arthritis, systemic lupus erythematosus, menopause, aging, women, autoimmune diseases

## Abstract

Rheumatoid arthritis and systemic lupus erythematosus (SLE) are autoimmune diseases that are commonly seen in the female population. Rheumatoid arthritis mainly consists of distal symmetrical deforming polyarthritis. SLE patients have immune complexes that damage the organs and systems of the body, and this can present with one or more symptoms including the characteristic malar rash, serositis, lupus nephritis, photosensitivity, and arthritis of large joints. The onset and progression of the diseases are affected by physiological processes that occur in the body such as menopause and aging. The studies used as evidence were found in the PubMed, ScienceDirect, ProQuest, Taylor & Francis Online, Wiley Online Library, Ovid, and Oxford Academic databases. By analyzing these studies, the effects of aging and menopause on rheumatoid arthritis and SLE were revealed. In relation to menopause and aging, it was found that there was a progression of disease in women who had rheumatoid arthritis. However, aging and menopause caused the progression of SLE to decrease in women. An earlier age of onset of menopause was correlated with an increased chance of developing rheumatoid arthritis and SLE. Furthermore, while some studies showed that a later onset of SLE caused an increase in the progression of the disease, other studies showed that a later onset of SLE led to a decrease in the progression of the disease. Due to the prevalence of rheumatoid arthritis and SLE in females, we believe that the effects of menopause, age, and other factors on these two diseases should be examined in future studies.

## Introduction and background

An autoimmune disease is a disorder in which a person’s body mistakenly attacks itself. It is estimated that approximately 5%-8% or 14-22 million people living in the United States have been diagnosed with autoimmune disease [1]. Of these, about 6.7 million, or 78.8%, are women [1]. In women, rheumatoid arthritis (RA) and systemic lupus erythematosus (SLE) are among the clinically most important autoimmune diseases [2]. The typical symptoms present in women with rheumatoid arthritis are stiffness and symmetrical swelling of joints [3]. Women with SLE often present with a malar rash on the face, photosensitivity, and Raynaud's phenomenon [4]. Mainstay therapy for rheumatoid arthritis consists of corticosteroids, non-steroidal anti-inflammatory drugs (NSAIDS), disease-modifying anti-rheumatic drugs (DMARDs) such as methotrexate, and biologic therapies, while those for SLE are corticosteroids, NSAIDS, antimalarials, immunosuppressives, and biologics [3,4]. RA and SLE have many articular and extra-articular complications that can involve the neurological, renal, hematological, gastrointestinal, cardiac, and pulmonary systems [3,4]. Though rheumatoid arthritis and SLE generally occur throughout a woman’s lifetime, they are undoubtedly affected by natural processes within the female body. As a woman ages, her ability to reproduce inevitably declines. The end of the reproductive period in women marks the beginning of many systemic changes within the body.

Menopause refers to the natural cessation of menses in a woman. Women who are affected by menopause are usually between 45 and 60 years [5]. Menopause is a direct result of the loss of ovarian sensitivity to gonadotropin stimulation. It is diagnosed by measuring the body's hormonal levels, such as follicle-stimulating hormone (FSH). When FSH levels are consistently high and a female has not menstruated for at least one year, she has reached menopause. The effects of this process lead to a woman undergoing many changes in her body due to the decrease in estrogen and progesterone. This decrease in hormonal levels can lead to osteoporosis, hot flashes, vaginal dryness, psychological changes such as mood disorders, and cardiovascular disease [5]. Medical therapy for menopause includes hormone replacement therapy, which consists of estrogen and progesterone for women who still have a uterus and only estrogen for those women who do not have a uterus [5]. Various modalities have been recognized to treat the symptoms associated with menopause, such as using estrogen for vaginal dryness, medications for osteoporosis, and low-dose antidepressants for mood disorders [6].

The deficiency of estrogen after menopause affects multiple systems in the body [7]. However, the influence of estrogen withdrawal on the immune system is not well understood [7]. As stated above, autoimmune diseases such as rheumatoid arthritis and SLE are more prevalent among women than men, so it is assumed that estrogen plays a significant role in their development [7]. Therefore, due to the declining estrogen levels associated with menopause, the relationship between menopause and the onset and progression of rheumatoid arthritis and SLE deserves further study.

Though menopause is a potential contributor to autoimmune diseases in women, increasing age could also be a contributor. As women get older, they are not only impacted by menopause but a variety of age-related physiological changes also impacts them. It is well known that these changes lead to a decline in overall health, which means the female body is less capable of overcoming adversity. In the United States and other countries worldwide, the incidence of many autoimmune diseases has risen steadily in recent years [7]. This increase has mirrored the global increase in female life expectancy [7]. Due to an ever-aging female population and the apparent predominance of autoimmune diseases in women, it is also essential to investigate the effects of aging on the emergence and advancement of rheumatoid arthritis and SLE. In this article, we will explore the influence of aging and menopause on the onset and progression of SLE and rheumatoid arthritis in women.

The articles used in this literature review were found in the PubMed, ScienceDirect, ProQuest, Taylor & Francis Online, Wiley Online Library, Ovid, and Oxford Academic databases. Articles published more than 25 years ago were not used in this paper. Additionally, every article was selected based on its relevance and detail. Keywords such as menopause, rheumatoid arthritis, systemic lupus erythematosus, aging, autoimmune diseases, and women were all used when selecting articles to cite.

## Review

Relationship between rheumatoid arthritis and menopause

Rheumatoid arthritis presents as swelling and tenderness in multiple joints located symmetrically in the body. Because of the higher prevalence of this disease in women compared to men, it is crucial to understand the relationship between female hormones such as estrogen and rheumatoid arthritis [3]. Engdahl et al. conducted a study on whether estrogen (E2) affected the immunoglobulin G (IgG) sialylation [8]. The researchers analyzed the IgG sialylation of postmenopausal (ovariectomized) mice and postmenopausal women between the ages of 45 and 65 [8]. It was found that E2 significantly increased Fc sialylation, which implied that E2 treatment induces anti-inflammatory effector functions [8]. Al-Salman reinforced the role of estrogen deficiency in the progression of rheumatoid arthritis [9]. In this study, 96 non-pregnant women with rheumatoid arthritis were divided into three groups: women of reproductive age (18-45 years), premenopausal women (46-53 years), and postmenopausal women (54-70 years) [9]. For each female participant, the serum concentrations of 17β-estradiol2 (17β-E2), interleukin-1β (IL-1β), and anti-cyclic citrullinated peptide were assessed by enzyme-linked immunosorbent assay [9]. Al-Salman found that 17β-E2 deficiency led to an increase in the IL-1β cytokine [9]. IL-1β is one of the most critical pro-inflammatory cytokines related to the pathogenesis of rheumatoid arthritis [9]. Like the previous studies, Mollard et al. also linked lower hormone levels during menopause to the advancement of rheumatoid arthritis [10]. To corroborate their claim, the researchers compared functional status in premenopausal and postmenopausal women with rheumatoid arthritis [10]. A Health Assessment Questionnaire (HAQ) was used to measure the functional status of premenopausal versus postmenopausal women who had rheumatoid arthritis [10]. HAQ is a three-point measure of functional status with 0 referring to no disability and three referring to severe disability [10]. The results of this questionnaire showed that, on average, the HAQ score for postmenopausal women is 0.68 higher than the HAQ score for premenopausal women [10]. Moreover, transitioning through menopause led to a 0.28 increase in HAQ scores, signifying increased disability [10].

Overall, these studies suggest that lower estrogen levels are associated with a progression of rheumatoid arthritis. Two of the three studies found a direct correlation between estrogen and the immune system. Engdahl et al. proved that estrogen has an anti-inflammatory effect by significantly increasing Fc sialylation [8]. The lack of estrogen during menopause causes the immune regulation by Fc sialylation to be eliminated. It ultimately leads to an increased chance for the progression of rheumatoid arthritis to be present in postmenopausal women between the ages of 45 and 65 [8]. The study performed by Al-Salman concluded that 17β -E2 deficiency increased pro-inflammatory cytokines such as IL-1β [9]. Pro-inflammatory cytokines are known for worsening the overall presentation of rheumatoid arthritis by further damaging the joints in the body. The functional status of premenopausal women and postmenopausal women was evaluated by Mollard et al., which proved that women with rheumatoid arthritis during menopause had a worse disability of function compared to women with rheumatoid arthritis during pre-menopause [10]. It is important to recognize that the results may have been influenced by longer disease duration and the degenerative processes associated with rheumatoid arthritis. Nevertheless, the impacts of estrogen deficiency due to menopause should not be dismissed.

Relationship between systemic lupus erythematosus and menopause

SLE is known for affecting multiple systems of the body, such as the kidney causing lupus nephritis, the joints causing damage to cartilage, and the skin causing the characteristic malar rash on the face and raised red patches throughout the body. SLE is usually first diagnosed during a woman’s reproductive years of life between 15 and 44 years [4]. Due to this fact, it is vital to understand the effect of hormone levels on the diagnosis and presentation of SLE. Mok et al. conducted a study focused on the rate and severity of disease exacerbation before and after menopause [11]. The study included a comparative analysis between a group of postmenopausal SLE patients who had disease onset before menopause and a group of patients who had a postmenopausal onset of SLE [11]. The researchers found that flares decreased significantly in postmenopausal patients who were first diagnosed with SLE before menopause [11]. Furthermore, the rates and magnitude of flares in previously diagnosed postmenopausal SLE patients were similar to those of postmenopausal onset SLE patients [11]. Bove supports this finding in his study, which summarizes the relationship between reproductive aging and how it affects autoimmune diseases [12]. According to this study, longitudinal studies that followed female subjects found that there was a decrease in the frequency of flares after the onset of menopause in patients with SLE [12]. It was also reported that there was a decrease in the SLE disease activity index [12]. On the contrary, it was concluded that there was a more significant damage accrual in affected organs from individual flares [12]. Khafagy et al. studied the direct relationship between exogenous estrogen through hormone therapy and its effect on the progression of SLE [13]. This study performed a systematic review to assess the impact of hormone therapy on the occurrence of flares in menopausal patients with SLE [13]. According to the results and conclusion of the study, SLE patients who use hormone therapy may have an increased risk of developing mild/moderate flares [13]. No association was found between severe flares and hormone therapy [13].

Two of these studies suggest that menopause reduces the overall severity of SLE. The decreased frequency of flares demonstrates this after menopause in women who have SLE. Mok et al. concluded that flares occur less frequently and less severely in postmenopausal women [11]. According to the study, this could be linked to estrogen deficiency during menopause or other factors such as disease duration or cytotoxic treatments [11]. The researchers suggest that further studies need to be conducted to determine whether a lack of estrogen directly affects the occurrence of flares and disease progression in women who have SLE [11]. Khafagy et al. concluded that the effect of hormone therapy on SLE might lead to a higher risk of mild/moderate flares [13]. Hormone therapy is meant to combat estrogen deficiency, so the potential for more mild/moderate flares suggests that estrogen deficiency alleviates the exacerbations associated with SLE [13]. Unlike the previous studies, Bove found both positive and negative effects of menopause on women with SLE [12]. Although it was reported by this study that there was a decrease in the frequency of flares, it was also reported that there was greater accrual damage in affected organs from individual flares [12]. Though the first two studies point to the positive impact of menopause on women with SLE, the conclusions made by Bove highlight the need for further studies on the relationship between female hormones and the progression of SLE [12].

Relationship between rheumatoid arthritis and age

The presentation of rheumatoid arthritis has been assessed in both premenopausal and postmenopausal women. It is crucial to understand that the onset and progression of rheumatoid arthritis during the reproductive period and beyond can be connected to the simple aging process in the female body. Kuiper et al. studied a cohort of patients consisting of both men and women [14]. The patients were followed for six years, and different factors were studied such as disease activity, radiographical joint destruction, and physical disability [14]. The results of the study noted that rheumatoid arthritis at a higher age of presentation leads to increased severity of disease as measured by the disease activity score (DAS), radiographic joint destruction (RJD), and the HAQ score [14]. Pawlowska et al. measured the disease activity in individuals compared to the age of onset of rheumatoid arthritis [15]. The expression of activation markers on CD4+ T cells was measured along with age at onset of disease and the disease activity in individuals [15]. The patients were divided into two groups with individuals who have an earlier onset of rheumatoid arthritis and individuals with a later onset of rheumatoid arthritis [15]. Those who had a later age of onset defined as more than 60 or 65 years had an increased CRP and ESR levels and D28 compared to those who had an earlier onset of the disease characterized as middle-aged [15]. Camacho et al. studied the relationship between age and functional disability in patients with a recent onset of inflammatory polyarthritis [16]. They concluded that older age at symptom onset caused an increasing steep progression of disability [16]. Patients with an early onset of symptoms had a steady progression over time [16].

Along with age, it is also of high importance to consider the postmenopausal state of women and age together as factors contributing to the onset, severity, and progression of rheumatoid arthritis. Beydoun et al. concluded in a study that women who enter menopause before the age of 40 are more likely to have rheumatoid arthritis following menopause [17]. However, age at menarche and history of pregnancy may not account for the development of the disease. Similar to Beydoun et al., Wong et al. also concluded that an earlier age of onset of menopause is associated with seropositivity in women with early rheumatoid arthritis [18]. Kobak et al. compared disease progression and presentation in those who had a young age at onset to those who had an elderly age at onset [19]. Results showed that compared to the young onset, the elderly onset of rheumatoid arthritis was characterized by a higher frequency of acute onset with accompanying constitutional symptoms, more frequent involvement of large joints, and a decreased frequency of rheumatoid factor positivity [19].

According to a majority of studies, age alone as a factor is related to disease severity. Kuiper et al. demonstrated that a higher age of presentation was directly correlated with an increase in the severity of disease [14]. Pawlowska et al. reported an increase in disease severity as a person ages due to the increase in activation of CD4+ markers, and Kobak et al. reported an increase in disease severity when the onset of the disease occurs in the elderly [15,19]. Camacho et al. found that the older age of onset caused further progression of the disease at a higher rate compared to the middle age of onset [16]. These studies convey that as a person ages, there is a worsening of the presentation and progression of rheumatoid arthritis. The link between early onset of menopause and postmenopausal rheumatoid arthritis was highlighted by Beydoun et al. and Wong et al. [17,18]. Both studies concluded that an earlier age of onset of menopause is associated with postmenopausal rheumatoid arthritis [17,18]. The increased chance of developing postmenopausal rheumatoid arthritis reinforces the connection between age and rheumatoid arthritis.

Relationship between systemic lupus erythematosus and age

SLE is typically seen in the younger female population. The time of initial diagnosis of SLE in women is most commonly during a woman’s reproductive years of life [4]. Among the various factors that contribute to the onset and progression of SLE, age should not be underestimated. Costenbader et al. performed a prospective cohort study on a total of 238,308 women who were both of older age and younger age [20]. Of the women who were studied, there were 262 reported incident cases of SLE [20]. It was found that early age of onset of menopause along with other factors such as oral contraceptive use, early age at menarche, surgical menopause, and postmenopausal use of hormones contributed to an increased chance to develop SLE [20]. Chen et al. compared the risk of mortality between a group of juvenile-onset, late-onset, and adult-onset SLE patients [21]. The researchers used a retrospective cohort study on previous data that was collected from the National Health Insurance Research Database of Taiwan between 2001 and 2004 [21]. Results of this study showed that female patients with a late onset of SLE had a higher risk of mortality compared to female patients with an adult-onset of SLE [21]. Both groups also had comorbidities [21]. Gen-Min et al. supported the evidence of age-related differences in the presentation of SLE by concluding in a study that adult-onset SLE is associated with a higher risk for lupus-related pulmonary involvement compared to childhood-onset SLE [22]. This was concluded after evaluating a longitudinal study by Hersh and colleagues [22]. Cildag et al. compared disease activity between late-onset SLE and adult-onset SLE [23]. Patients were divided into two groups based on their age of onset of SLE [23]. Those who had a late onset of SLE had less disease activity, less organ involvement, and less renal involvement compared to those with adult-onset and juvenile-onset SLE [23]. Gompel et al. reviewed a compilation of studies, and the researchers concluded that disease duration is related to the disease onset and severity [24]. One of these studies was a French study conducted on 47 patients who were diagnosed with SLE at or after the age of 50 and on 114 patients who were diagnosed with SLE at an age younger than 50 [24]. It was found that compared to the premenopausal group of women, the postmenopausal group of women had a less frequent occurrence of arthritis, malar rash, and nephropathy and a more frequent occurrence of serositis, lung involvement, and Sjogren syndrome [24]. Another compilation study was a Canadian study that compared SLE in premenopausal women, postmenopausal women, and women in the menopause transition phase [24]. This study concluded that vasculitis, proteinuria, rash, pericarditis, and the presence of anti-DNA antibodies were higher in premenopausal women compared to postmenopausal women [24]. The researchers clearly made the claim that it was the duration of disease activity not the postmenopausal status that led to an overall decrease in disease activity [24].

Most of these studies suggest that there is a relationship between age and the onset and progression of SLE. Constenbrader et al. found that early age of onset of menopause and menarche is correlated with an increased risk of developing SLE [20]. Two of these studies compared the effect of disease activity on the earlier age of onset to a later age of onset of SLE. Chen et al. and Gen-Min et al. came to a similar conclusion in different ways [21,22]. Chen et al. stated that there is an increase in mortality with a later onset of disease, and Gen-Min et al. claimed that the childhood onset of SLE causes a lower risk in the pulmonary involvement of the disease as compared to the adult onset of SLE [21,22]. Cildag et al. concluded that the late onset of SLE correlated with a decrease in the disease progression of SLE as compared to an earlier onset of SLE [23]. Gompel et al. proclaimed that elongated disease duration rather than a postmenopausal state in women caused a decrease in disease activity [24].

Table 1 summarizes the relevant findings of each study included in the review section by stating whether menopause or age caused an improvement or exacerbation of the disease.

**Table 1:** Summary of Study Findings 17β-E2: 17β-estradiol2; IL-1β: interleukin-1β; SLE: systemic lupus erythematosus.

Factors	Studies	Disease	Improvement/Exacerbation
Menopause	Engdahl et al. [8]	Rheumatoid arthritis	Exacerbation: Estrogen deficiency caused immune regulation by Fc sialylation to be eliminated.
	Al-Salman [9]	Rheumatoid arthritis	Exacerbation: 17β-E2 deficiency resulted in an increase in pro-inflammatory cytokines such as IL-1β.
	Mollard et al. [10]	Rheumatoid arthritis	Exacerbation: Postmenopausal women had worse disability of function compared to women with rheumatoid arthritis during premenopause.
	Mok et al. [11]	SLE	Improvement: Flares occur less frequently and less severely in postmenopausal women.
	Bove [12]	SLE	Exacerbation: Greater accrual damage in affected organs from individual flares. Improvement: Decrease in the frequency of flares.
	Khafagy et al. [13]	SLE	Exacerbation: Hormone therapy during menopause may lead to a higher risk of mild/moderate flares.
Age	Kuiper et al. [14]	Rheumatoid arthritis	Exacerbation: A higher age of presentation was directly correlated with an increase in the severity of disease.
	Pawlowska et al. [15]	Rheumatoid arthritis	Exacerbation: Increase in disease severity as a person ages due to the increase in activation of CD4+ markers.
	Camacho et al. [16]	Rheumatoid arthritis	Exacerbation: Older age of onset caused further progression of the disease at a higher rate.
	Beydoun et al. [17]	Rheumatoid arthritis	Exacerbation: Earlier age of onset of menopause is associated with postmenopausal rheumatoid arthritis.
	Wong et al. [18]	Rheumatoid arthritis	Exacerbation: Earlier age of onset of menopause is associated with postmenopausal rheumatoid arthritis.
	Kobak et al. [19]	Rheumatoid arthritis	Exacerbation: Increase in disease severity when the onset of disease occurs in the elderly.
	Costenbader et al. [20]	SLE	Exacerbation: Early age of onset of menopause and menarche are correlated with an increased risk of developing SLE.
	Chen et al. [21]	SLE	Exacerbation: Increase in mortality with a later onset of disease.
	Gen-Min et al. [22]	SLE	Exacerbation: The childhood onset of SLE causes a lower risk in the pulmonary involvement of the disease as compared to the adult onset of SLE.
	Cildag et al. [23]	SLE	Improvement: Late onset of SLE correlates with a decrease in the disease progression of SLE as compared to an earlier onset of SLE.
	Gompel et al. [24]	SLE	Improvement: Elongated disease duration rather than a postmenopausal state in women caused a decrease in disease activity.

## Conclusions

The onset and progression of autoimmune diseases that are more common in women such as rheumatoid arthritis and SLE may be correlated with aging and menopause. Multiple articles have indicated that a decrease in estrogen is related to the disease progression of rheumatoid arthritis. With this relationship, aging as a coexisting factor affecting the progression of rheumatoid arthritis is also raised into question. A majority of studies found that similar to the negative effects of estrogen deficiency on rheumatoid arthritis in women, aging also worsens rheumatoid arthritis. Multiple studies show that a later age of onset caused disease progression at an increased rate as compared to a middle age of onset. Female patients suffering from SLE have a decrease in the severity of disease in the form of flares after menopause as highlighted by many articles. Articles related to SLE have also found that the age of onset of SLE in women is related to the disease progression of SLE and as a woman ages, there is a decrease in the progression of disease. In the future, the influence of menopause and aging on the onset and progression of rheumatoid arthritis and SLE should be further investigated by researchers. Moreover, other factors impacting rheumatoid arthritis and SLE in women should also be explored.
